# Dynamic Modulation of DNA Replication and Gene Transcription in Deep-Sea Filamentous Phage SW1 in Response to Changes of Host Growth and Temperature

**DOI:** 10.1371/journal.pone.0041578

**Published:** 2012-08-01

**Authors:** Huahua Jian, Jun Xu, Xiang Xiao, Fengping Wang

**Affiliations:** 1 School of Life Sciences, Xiamen University, Xiamen, China; 2 State Key Laboratory of Microbial Metabolism and School of Life Sciences and Biotechnology, State Key Laboratory of Ocean Engineering, Shanghai Jiao Tong University, Shanghai, PR China; 3 Key Laboratory of Systems Biomedicine, Ministry of Education, Shanghai Jiao Tong University, Shanghai, PR China; University of Illinois at Chicago College of Medicine, United States of America

## Abstract

Little is known about the response of deep-sea virus and their relationship with their host towards environmental change. Although viruses are thought to play key roles in the deep-sea ecological evolution and biogeochemical cycling, these roles are yet to be defined. This study aims to delineate the relationship between a deep-sea filamentous phage SW1 and its host *Shewanella piezotolerans* (*S. piezotolerans)* WP3, and their response towards temperature change. The copy number of SW1’s replicative form (RF-) DNA and single-stranded (ss-) DNA along the different growth phases of WP3 were quantified at 20°C and 4°C, respectively. The copy number of SW1 RF-DNA was found to be temperature and growth phase-dependent, while the ssDNA of SW1 was only produced at 4°C. This is the first report showing low-temperature dependence of phage DNA replication. The transcription of SW1 key genes *fpsA* and *fpsR* were also found to be induced at low temperature during all the monitored growth periods of WP3. Additionally, the transcription of SW1 was found to be induced by cold-shock while its DNA replication was not changed. Our data demonstrates a dynamic change of virus DNA replication and transcription in accordance with host growth, and the low temperature adapted mechanisms for SW1 activities in the deep sea. This low temperature adapted deep-sea virus-bacterium system could serve as an ideal model to further study the mechanism and relationship of deep-sea virus-bacteria ecosystems.

## Introduction

Deep ocean covers approximately 60% of the earth’s surface and is the largest ecosystem on earth. It was believed that viruses, especially bacteriophage, play an important role in deep-sea metabolism, global biogeochemical cycles and the overall functioning of the largest ecosystem of our planet [1]. The temperature in this environment is about 2∼4°C with the exception of hydrothermal vents [2]. This has led us to believe that the overwhelming majority of phage in deep-sea is “cold-active” [3]. As few cold-active phages have been isolated from marine sediments, very little is known about their basic biological properties, how they respond to environmental change, and how this affects their relationship with hosts [3], [4], [5].

The deep-sea bacterium *Shewanella piezotolerans* WP3 (hereafter referred to as WP3) which harbors a filamentous phage SW1, was isolated from the West Pacific sediment at a depth of 1914 m [6], [7]. The temperature range for WP3 growth is 0–28°C with the optimum growth temperature being 20°C. No active phage particles were isolated from the WP3 cultures incubated above 20°C and more phage production was observed at 4°C than at 10°C or 15°C [8]. This suggests that SW1 is the first cold-active filamentous phage ever documented.

**Figure 1:**
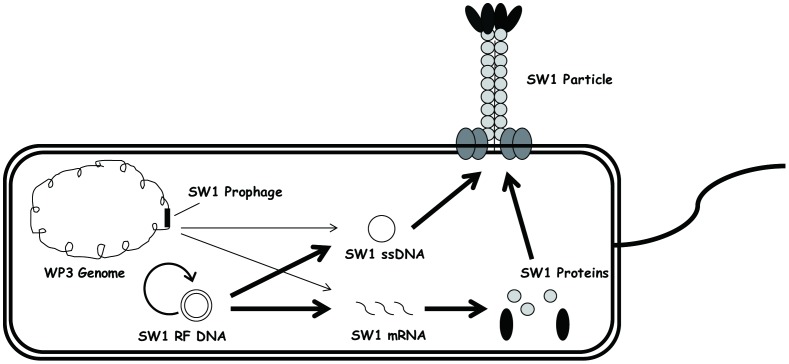
Proposed schematic cartoon showing the different forms of SW1 in the WP3 cell. The lysogenic WP3 cell contains 3 different forms of SW1 DNA: The prophage integrated in the bacterial genome, the double-strand replicative form DNA (RF DNA) and the single-strand DNA (ssDNA) in the cytoplasm. Like the plasmid, RF DNA possesses the capability of self-replication. Both of the prophage and RF DNA serve as template for ssDNA and mRNA production. This cartoon is proposed based on our previous data [8], [15] and knowledge from well-characterized filamentous phages such as Ff and CTXΦ [9], [10], [11], [14].

Filamentous phage constitute a large family of bacterial virus that infect a variety of Gram-negative bacteria [9]. In general, the virion is a flexible rod with a sheath of several thousand identical capsid proteins outside, and a single-stranded circular DNA at the core [10]. The most well studied filamentous phages are Ff and CTXΦ which infect *E. coli* and *Vibrio cholera*, repectively. Ff exist in the host as a plasmid which is called replicative form DNA (RF DNA) while CTXΦ integrated into bacterial genome as a prophage [9]. Both the integrated prophage and the RF DNA of filamentous phage have been demonstrated able to produce ssDNA and transcripts [9], [11]. The replication of filamentous phage DNA can be divided into minus and plus strand synthesis. Minus strand synthesis converts the viral ssDNA to a dsDNA, and is initiated by an RNA primer which is synthesized by host RNA polymerase holoenzyme containing the σ^70^ subunit. The replication of the plus strand proceeds via a rolling-circle mechanism on the double-stranded RF DNA template. Phage encoded initiator protein plays central roles in this process. It binds to the plus-strand origin which harbours signals for initiation and termination, and then a single-strand break was introduced. As the initiation of phage DNA replication is a key step for phage DNA replication, it’s thus under strict control [12], [13], [14]. Our previous genome sequencing project demonstrated that the genome of SW1 integrates into its host’s chromosome flanked by 13 base pair (GAATGCGCACAAT) direct repeats, and can also maintained in the cytoplasm as RF DNA [15] (Figure 1). Thus far, it is not clear how environmental change such as temperature shift influences the DNA replication and gene transcription of SW1.

Temperature is one of the major stressors that microorganisms endure. To date, cold-shock response and adaptation of some mesophilic and psychrophilic bacterial cells have been extensively studied [16], [17], [18], [19]. However, the influences of temperature changes on the activity of bacteriophage have not been extensively investigated. Temperature has previously been found to affect the lysis-lysogeny mechanism of λ phage and phage DNA replication which was completely blocked at low temperature (20°C) [20], [21], [22]. It still remains unclear if these kind of temperature influences occur in other bacteriophage. Moreover, it is completely unknown if and how temperature affects the behavior of temperate phage such as the filamentous phage. Here, we investigated the influences of temperature change on the response of the cold-active deep-sea filamentous phage SW1 and its relationship with the growth of its host cells. The SW1 DNA copy number and relative mRNA levels were measured during the whole life cycle of WP3. DNA replication and gene transcription of SW1 were found both to be related to host growth and temperature. Moreover, cold shock was found to induce the gene transcription of SW1, but have no influence on SW1 DNA replication. Elucidating the mechanisms implicated in these changes would be fundamental to understand the behavior and roles of deep-sea cold-active phages and phage-host ecosystems towards environmental change.

## Results

### Amplification Efficiency of Primer Sets for Copy Number Determination

DNA of filamentous phage SW1 can exist as 3 forms in the host WP3 cells (Figure 1): integrated prophage, replicative double-stranded DNA (RF DNA) and single-stranded DNA (ssDNA). The RF DNA of SW1 is self-replicating similar to plasmids (pSW1) and is the template for ssDNA production. The copy number of RF DNA and ssDNA form of SW1 was determined by Q-PCR as described in the materials and methods.

The quantitative standard plasmid pSW3 was constructed based on the pSW2 which contain over 60% sequence of SW1 [23] (Table 1). We analyzed the WP3 genome and selected a single-copy specific *pepN* gene as the target gene (Table 2). The *pepN* gene encodes aminopeptidase N, which was found constitutively transcribed in different culturing conditions [24]. *pepN* was also used for the endogenous reference gene for the relative quantification of SW1 gene expression [8], [15], [23], [25], [26].

**Table 1:** Bacterial strains and plasmids used in this study.

Strain or plasmid	Relevant genotype	Reference or source
*E. coli* strain		
WM3064	*thrB1004 pro thi rpsL hsdS lacZ*ΔM15 RP4-1360 Δ(*araBAD*)567 Δ*dapA1341*::[erm pir(wt)]	[38]
DH5α	Φ80d*lacZ*ΔM15 (*lacZYA-argF*) U196 *recA1 hsdR17 deoR thi-1 supE44 gyrA96 relA1*/λpir	Lab stock
*S. piezotolerans* WP3 strains		
WP3-WT	Deep-sea bacterium	Lab stock
Plasmids		
pSW1	Replicative form of SW1	Lab stock
pSW2	Low-temperature expression vector based on pSW1	Lab stock [23] (unpublished data)
pSW3	pSW2 containing the single copy fragment of *pepN*	This work

**Table 2:** Primers used in this study.

Primer name	Sequence (5′-3′)	Description	
pepNAmpFor	GTTAACGCGTTATGAAATAGAGCGCCCACC		pSW3 construction
pepNAmpRev	TTCTCTCGAGTGTTCGTCCTGAGGAAACAGT		pSW3 construction
SW1RFRT For	CACGCCATACGTTAATGAGTCTCT		qPCR
SW1RFRT Rev	GACGGCCAGTATTCAACATAACAT		qPCR
pepNRT For	TTAAGGCAATGGAAGCTGCAT		qPCR
pepNRT Rev	CGTCTTTACCCGTTAATGATACGA		qPCR
*fpsA*RT For	GGCCACAACGCTACAATGG		qPCR
*fpsA*RT Rev	TGTTGCGCCTTTTCCTTAGCT		qPCR
*fpsR*RT For	AACCTAGAAACACTTGTCGCTATATCC		qPCR
*fpsR*RT Rev	TGCTCTCCAAAAACAATTTCATCT		qPCR
*cspA*RT For	GGCTTCATCACTCAAGACAATGG		qPCR
*cspA*RT Rev	AAGCGATTGCACGGAAATGT		qPCR

The standard curve for SW1RFRTFor/Rev and pepNRTFor/Rev, each ranging from 1×10^3^ to 1×10^7^ copies^−1^, are shown in Figure S1. Both curves were highly linear (R^2^>0.999) in the range tested. The slopes of the standard curves for the two primer sets were −3.699 and −3.649, respectively. From the slopes, amplification efficiencies of 86.326% and 87.635% were determined for SW1RF and *pepN* primer sets respectively. The efficiency values were used for quantification of DNA copy number. Amplification specificity was validated by melt curve analysis. Both primer sets showed a single melting peak indicating that non-specific PCR products were not detected in the analyzed temperature range.

### Copy Number of SW1 RF DNA is Related to the Growth Phase and Temperature

**Figure 2:**
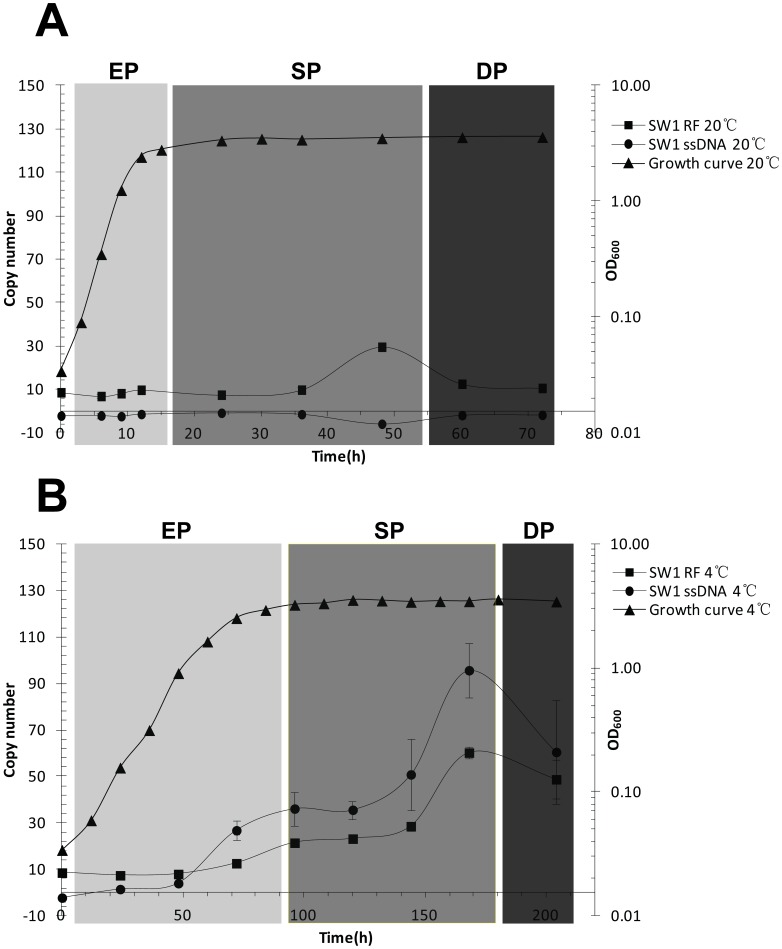
SW1 RF DNA and ssDNA copy number variations over the life cycle of WP3 at different temperatures. The same amount of WP3 culture was inoculated in 2216E medium and then incubated at 20°C (A) and 4°C (B). The samples were taken from the cultures at different time point and the DNA copy number was determined. The data shown above represents two independent experiments and the error bars indicate standard deviations from triplicate assays. The different growth phases were represented with different colors. EP: Exponeitial phase, SP: Stationary phase and DP: Decline phase.

A growth curve assay for WP3 was performed at 20°C (optimal growth temperature) and 4°C (in-situ deep-sea environmental temperature). Eight samples at different time points covering the whole life cycle of WP3 were collected at the two temperatures (Figure 2). Total DNA was extracted and the RF DNA copy number (RCN) was quantified. The data showed that during the exponential growth phase, copy number was approximately 10 at both temperatures. Once the host entered the stationary growth phase, SW1 RCN at 4°C began to increase while it was stable at 20°C. At mid-stationary phase, the copy number at 4°C was 30, 3-fold more than that at 20°C. There is a peak at the late-stationary phase of RCN value at both temperatures. The RCN was 29.6 and 60.2 at 20°C and 4°C, respectively. The RCN decreased when the cell entered the decline phase with a value similar to the exponential phase at 20°C. These data indicated SW1 RF DNA is higher in copy number at 4°C in contrast with 20°C for the majority of the life cycle, suggesting that DNA replication of filamentous phage SW1 is temperature-dependent. We also found an increase in RCN during the late-stationary phase at both temperatures, suggesting that some growth phase-related factors may play a role in this process.

### Production of ssDNA is Low Temperature Inducible

During the life cycle of filamentous phage, ssDNA production is coupled with phage particle assembly and maturation. Therefore, it is a key indicator of phage production. The production and quantity of SW1 ssDNA were assayed at both 20°C and 4°C during the growth of WP3 (Figure 2). There was no ssDNA produced at 20°C during the whole life cycle of WP3, while a significant amount of ssDNA was generated at 4°C except at the early-exponential growth phase of WP3 (Figure 2B). Interestingly, the copy number of ssDNA was similar to RF DNA at low temperature. The amount of ssDNA was increased with RF DNA when the cell entered stationary phase and the maximum value (47.8) was observed at the late-stationary phase. Subsequently, the copy number of ssDNA began to decrease (Figure 2B).

### Transcription of SW1 Genes

SW1 gene transcription during the growth of its host WP3 was investigated. Two key genes, *fpsA* and *fpsR*, were chosen as representatives. FpsA consists of 541 amino acids and shares the highest sequence identity with replication protein RstA (52%) of CTXΦ. FpsR contains 116 amino acids and its N-terminal peptide has a typical Cro/CI type helix-turn-helix DNA binding motif but lacks the putative peptidase domain, suggesting it is a transcriptional repressor but may not have the protein autoproteolysis function [8], [15]. As shown in Figure S2, the relative mRNA level of these two genes is significantly associated with temperature in all growth stages. The ratio of *fpsA* mRNA level between 4°C and 20°C was 2.98, 16.09 and 20.34 in mid-exponential phase, mid-stationary phase and decline phase, respectively. Similarly, the ratio of *fpsR* mRNA level between these two temperatures was 5.19, 14.34 and 9.38 in the above growth phases. The *fpsA* mRNA level continued to increase from the beginning of cell cultivation and peaked in the decline phase, while the total mRNA level of *fpsR* was more stable, with the lowest value observed in the late-exponential phase (Figure S2B).

**Figure 3:**
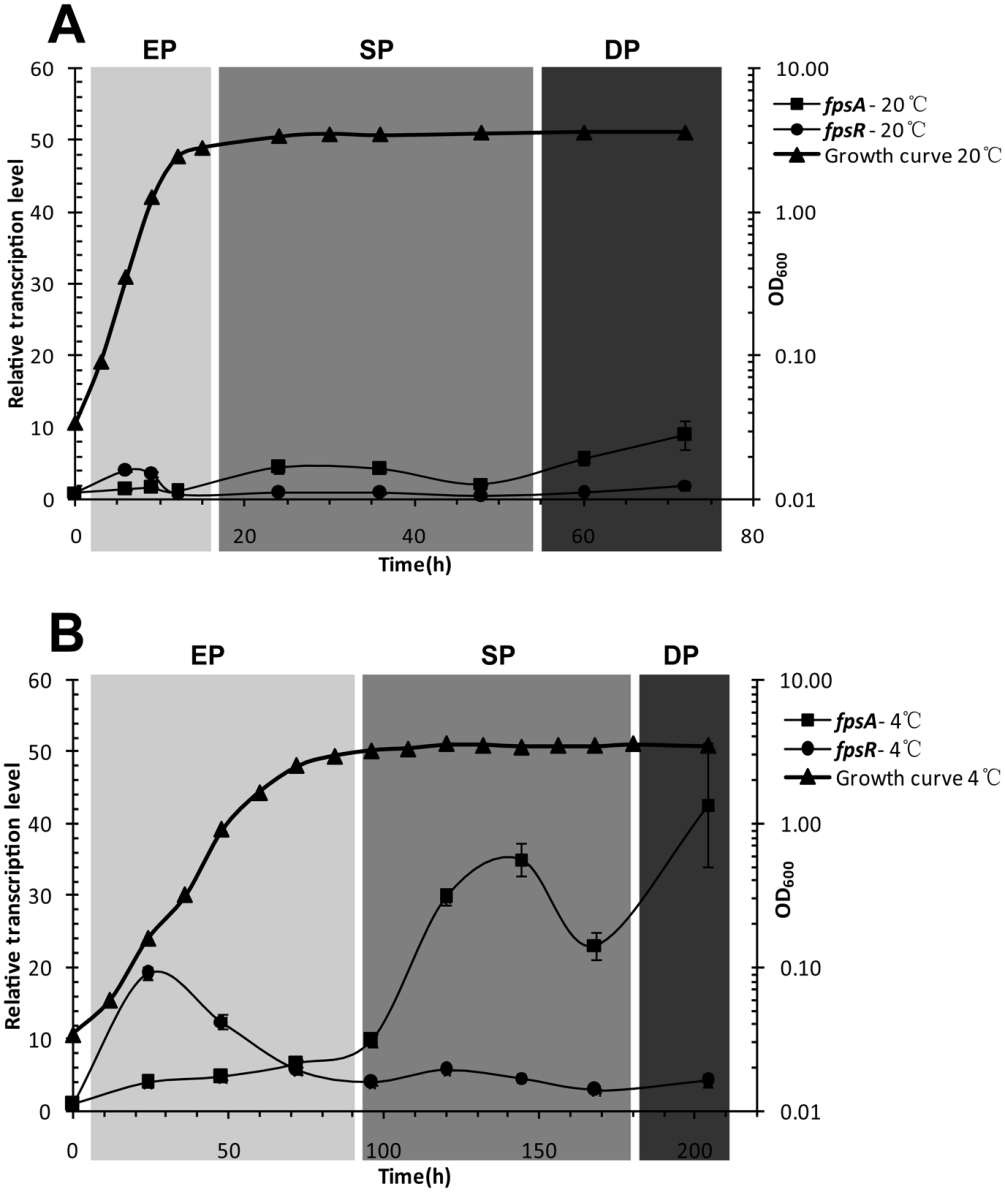
The relative transcription level (RTL) of *P_fpsA_* and *P_fpsR_* over the life cycle of WP3 at different temperatures. The same amount of WP3 culture was inoculated in 2216E medium and then incubated at 20°C (A) and 4°C (B). The RTL value were obtained by the calibration of the relative mRNA level of *fpsA* and *fpsR* with SW1 RF DNA copy number. The RTL of the two promoters at zero point were set as 1. The data shown above represents two independent experiments and the error bars indicate standard deviations from triplicate assays. The different growth phases were represented with different colors. EP: Exponeitial phase, SP: Stationary phase and DP: Decline phase.

As the copy number of SW1 RF DNA (phage replication) is related to growth phase and temperature (Figure 2), it is uncertain if the observed change of mRNA quantity is the effect of DNA copy change or if the transcription level per gene is also affected. The relative transcription level (RTL) of *fpsA* and *fpsR* was calculated by calibrating the mRNA levels with RCN of SW1, respectively as shown in Figure 3. As a whole, The RTLs of *fpsA* and *fpsR* are significantly higher at 4°C than those at 20°C, demonstrating that SW1 gene transcription is more active at lower temperatures. The RTL of *fpsA* and *fpsR* also correlated with host growth phase with the highest RTL value of *fpsA* and *fpsR* in the cell decline phase and middle exponential phase, respectively. In contrast, the RTL of *fpsR* increased at the initial stage and decreased after the mid-exponential phase while the RTL of *fpsA* started to increase. The transition between exponential phase and stationary phase is a crucial time point because the RTL of the two genes was almost equal (1.05 and 0.72 at 20°C, 6.58 and 5.66 at 4°C) during this time. After this time point the RTL of *fpsR* maintained a low value and the RTL of *fpsA* increased markedly. Moreover, a decrease in *fpsA* RTL was observed in the late-stationary phase which corresponds with the peak of RF DNA copy number in the same growth phase.

### SW1 Gene Transcription is Cold-shock Inducible

**Figure 4:**
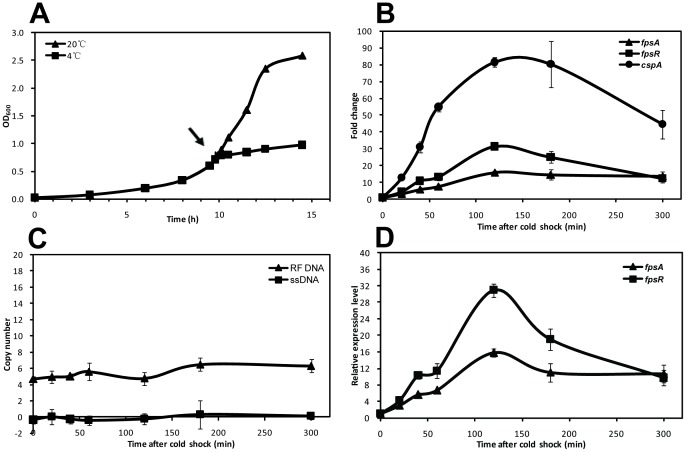
Effects of cold shock on SW1 DNA replication and gene expression. (A) Growth of *S. piezotolerans* WP3 at 20°C and after cold shock to 4°C. The arrow indicate the time point of cold shock (OD_600_ = 0.598). (B) Transcription level of *cspA*, *fpsA* and *fpsR* upon cold shock. The transcription level of these genes before cold shock (zero point) was set as 1. (C) Determination of copy numbers of SW1RF DNA and ssDNA after cold shock. (D) Relative transcription levels of *fpsA* and *fpsR* upon cold shock. As for the different copy number of SW1 RF DNA, the transcription levels of *fpsA* and *fpsR* were standardized to the copy number of transcriptional templates (RF and prophage of SW1). The transcription level of the two genes at zero point was set as 1. Standard deviations are indicated by error bars from triplicate assays. All of the data shown represent two independent experiments.

The response of WP3 growth and SW1 (replication and transcription) toward abrupt temperature change (e.g. cold shock) were investigated using the methods described above. The growth of WP3 paused immediately upon cold shock (a temperature shift from 20°C to 4°C) and then resumed after a 3 h lag period (Figure 4A). The relative mRNA level of *fpsA* and *fpsR* were compared between cold-shocked WP3 cells and non treated WP3 cells. Of the 6 selected time points, the results showed that once the WP3 was transferred from 20° to 4°C, the relative mRNA amount of *fpsA, fpsR* and *cspA* were up-regulated 3.2, 4.4 and 12.8-fold after 20 min, respectively (Figure 4B). The peak of mRNA expression appeared at 120 min after cold-shock, and the relative mRNA expression of *fpsA* and *fpsR* increased 16.0 and 31.3-fold, respectively. At the same time, the transcription level of *cspA* was activated 81.4-fold indicating an intense cold-shock response.

To further clarify the effect of cold shock on the DNA replication or the transcription of SW1, the RCN of SW1 was determined as shown in Figure 4C. There were minimal changes in RCN and it remained stable at 4∼6 copies per cell and there is no ssDNA production measured. After calibrating the relative mRNA quantity of *fpsA* and *fpsR* to SW1 RCN, the RTL of the transcripts after cold shock treatment could be obtained (Figure 4D). The transcription level of *fpsA* and *fpsR* at zero time point were set as 1, and then a 3 ∼ 15-fold and 4 ∼ 31-fold increase in transcription was observed, respectively. Our data delineate that the gene transcription of SW1 is cold-shock inducible, while its DNA replication is not affected by cold shock response.

## Discussion

In this study, the response of a filamentous phage SW1 towards temperature decrease and its relationship with its host WP3 was investigated. Filamentous phage can integrate into their host’s chromosome as prophage, and are also presented in the cytoplasm as RF DNA. Our initial results demonstrated that the copy number of RF DNA and ssDNA changed with temperature and host growth phase. The copy number of RF DNA was maintained at 10 at 20°C, increasing in the late-stationary phase. More RF DNAs were synthesized at low temperature, for example, during the mid-stationary phase; the copy number of RF DNA was 28.6 and 9.8 at 20°C and 4°C, respectively. ssDNA was only produced at 4°C and we did not detect ssDNA in the whole life cycle of WP3 at 20°C (Figure 2). These results coincide with our previous results where no phage particle was observed at above 20°C [8]. As phage usually uses host’s DNA replication apparatus to replicates its DNA and it is well known that multiple host factors participated in the initial phase of filamentous phage DNA synthesis, such as RNA polymerase and integration host factor (IHF) [14]. This indicates that temperature influences the initiation of SW1 DNA replication.

The “lysis or lysogeny” mechanism of phage λ is affected by temperature, and it was demonstrated that although lysogenization is efficient at low temperature (20–25°C), the lytic development of λ phage is completely inhibited at 20°C [20]. Further research shows that the increasing stability of CII is responsible for the cold-inhibition of λ phage DNA replication [21], [22]. Previous studies investigating heat shock and λ plasmid replication have indicated that heat-shock proteins have both positive and negative functions in the regulation of λ plasmid replication [27]. For instance, heat shock proteins DnaK, DnaJ and GrpE are absolutely necessary for initiation of DNA replication from *oriλ* [28], [29], [30], however, λ plasmid copy number (PCN) is decreased at 42°C relative to a lower temperature (30°C or 37°C) [31]. Our study is the first to show that phage DNA replication is induced at low temperature, and suggests a novel regulatory mechanism which is different from classic phage λ. Due to the low temperature in the deep-sea environment, we can speculate that the cold-dependent DNA replication of phage may be a universal phenomenon which is important for the ecological function of bacteriophage in the abyssal ecosystems.

Previous studies have reported the relative mRNA level of the SW1 gene is higher at 4°C than 20°C in the exponential phase [8]. Moreover our results suggest that the higher mRNA level at low temperature exists during the entire life cycle of WP3. After calibrating the mRNA level with copy number of RF DNA, we calculated the relative transcription level of SW1 key genes. The results showed that the transcription of pfsA and *fpsR* was more active at 4°C than 20°C (Figure S2 and 3). *fpsA* and *fpsR* transcription exhibited opposite patterns of variation. When the transcription of *fpsR* remains at a high level, the transcription level of *fpsA* is low and no RF DNA and ssDNA of SW1 was generated. Once the cell entered mid-exponential phase and the RTL of *fpsR* began to fall, the RLT of *fpsA* increase steadily. During the stationary and decline phase of WP3, RTL of *fpsR* remains at a low value while the RTL of *fpsA* is quite high. Besides that, the RF DNA and ssDNA are abundant when the RTL of *fpsR* is low. (Figure 2B and 3B). From the above findings, we speculated that FpsR is a functional repressor for SW1 regulation, because of the obvious inhibition on *fpsA* transcription and the replication of SW1 DNA.

It should be pointed out that host factors may play important roles in the regulation of SW1 DNA replication. Firstly, the divergence of RF DNA and ssDNA copy number between the two temperatures appeared at the late-exponential phase, where previously there was no significant difference. Secondly, the peaks of copy number were observed in the late-stationary phase under both culture conditions, this result matches the characteristics of “escape replication” [32], [33], [34]. During nutrient starvation the bacterium enters the decline phase and cannot reproduce. Hence, the status of the host can be sensed by the bacteriophage which prompts it to produce more particles to infect a new host. In this phase, the replication apparatus was activated and the ssDNA was generated in a large amount.

Besides the effect on SW1 DNA replication, the actions of growth phase related factors on gene transcription cannot be ignored. At 4°C, for example, from the mid to late-exponential phase, the RTL of *fpsR* decreased 3.4-fold while the RTL of *fpsA* increased 3.6-fold from early to mid-stationary phase. At 20°C, the fold changes are 5.5 and 3.9 for RTL of *fpsR* and *fpsA*, respectively (Figure 3). These data indicate that the transition is the impact of host growth phase rather than temperature regulation.

Overall, our results have shown that SW1 is induced by low temperature at both DNA replication and gene transcription level. Our results also demonstrate that the copy number and mRNA level of SW1 changed with the growth phase of WP3, which suggests that host sigma factors, such as stationary phase specific factor σ^38^, may participate in the regulation of SW1. The exact regulatory mechanisms for SW1 replication and transcription still need to be further investigated. Moreover, our results show that SW1 DNA replication and gene expression is cold-shock inducible, which means a rapid response of replication and transcriptional machinery to the abrupt temperature downshifts. Our data also revealed that the transcription strength of promoter of *fpsA* (P_A_) increased 3-fold in 20 min upon cold-shock, and 4-fold for promoter of *fpsR* (P_R_) (Figure 4)_._

In *E. coli*, the *cspA* mRNA contains a 159-bp 5′UTR which is a major determinant of temperature sensitivity [35], [36], [37]. Since the mRNA level of *fpsA* and *fpsR* both increased at low temperature or after cold-shock and the existence of long 5′UTR upstream of *P_A_* and *P_R_* (unpublished data), it is very likely that the extended half-life time of mRNA may be the reason for the increase in mRNA level of SW1 genes.

In conclusion, our results demonstrated, for the first time, the phage DNA replication is cold-dependent. The dynamic change of SW1 DNA replication and gene transcription in the host life cycle at different temperature implies a close connection between deep-sea bacterium and bacteriophage. Our data also delineated the combined action of environmental factors and inner regulatory systems on the phage activity. Further research will not only extend our understanding of host-phage interaction and its ecological significance, but also lay a solid foundation for the development of cold-active microbial cell machineries.

## Materials and Methods

### Strains and Growth Conditions

The bacterial strains and plasmids used in this study are listed in Table 1. *S. piezotolerans* WP3 was isolated from deep-sea sediment samples in our laboratory [6], [7]. In this study, WP3 was cultured in modified marine medium 2216E (5 g/L tryptone, 1 g/L yeast extract, 0.1 g/L FePO_4_, 34 g/L NaCl) aerobically with shaking at 200 rpm at different temperatures as indicated in the text. *E. coli* strain DH5α were incubated in Luria-Bertani (LB, 10 g/L tryptone, 5 g/L yeast extract, 10 g/L NaCl) media at 37°C. The Chloramphenicol was used at the concentration of 25 µg/mL.

### The Growth Curve and Sample Collection

The single clone of WP3 was inoculated into a 5 mL test tube and grown overnight. The culture was diluted 1000-fold in the same medium and was grown to late-exponential phase (OD_600_≈2.0), and then 100 µL culture was transferred into a 1000 mL shake flasks containing 200 mL medium and incubated at 20°C and 4°C respectively. During the life cycle of WP3, the samples were collected at different times and the OD_600_ was monitored with a spectrophotometer (SHIMADZU UV-2550). For the sample treatment, culture volumes of 1 mL were periodically removed from the shake-flasks and centrifuged for 30 s at the maxim speed. The cells were immediately frozen in liquid nitrogen for subsequent DNA and RNA extraction.

### Cold Shock Assay

Cells for cold shock response were prepared as described previously [38] with some modifications. Briefly, the WP3 strain was grown in 2216 marine broth at 20°C overnight and diluted into fresh 2216E medium. Cells were grown at 20°C to middle exponential phase (OD_600_ of 0.8), and part of the culture was harvested and used as a control (Zero point). The remainder was divided into two parts, one half was transferred to a 250-mL flask pre-chilled to 4°C and then incubated in a 4°C shaker for cold shock, the other half was cultured continuously in a 20°C shaker. Samples were harvested by centrifugation and immediately placed in liquid nitrogen at 0 (control), 20, 40, 60, 120, 180 and 300 min after a temperature downshift.

### Total DNA Isolation and S1 Nuclease Treatment

Genomic DNA isolation was performed following the procedures described by Sambrook *et al* [39]. Digestion of ssDNA was performed using S1 nuclease. Four Units were added to 500 ng of total DNA in a standard buffer solution (40 mM sodium acetate, 300 mM NaCl, 2 mM ZnSO4, pH 4.6), incubated at 37°C for 30 min and the enzyme was inactivated by heating at 70°C for 5 min.

### RNA Extraction and Reverse Transcription (RT)

Total RNA was isolated from the WP3 culture growing at different temperatures with TRI reagent-RNA/DNA/protein isolation kit (Molecular research center, Cincinnati, USA) according to the manufacturer’s instructions. The RNA samples were treated with DNase I at 37°C for 1 h to remove DNA contamination. The quantity and integrity of RNA was evaluated with a UV spectrophotometer (Nanodrop 2000c, Thermo Scientific) and agarose gel electrophoresis prior to the experiments. The purified RNA samples were used to synthesize cDNA with the RevertAid First Strand cDNA Synthesis Kit (Fermentas, Maryland, USA) following the manufacturer’s instructions.

### Construction of the Standard Plasmid pSW3

The PCR product of *pepN* gene was amplified from WP3 genomic DNA and purified using the E.Z.N.A Cycle-Pure kit I (Omega Bio-Tek Inc, Norcross, USA) according to manufacturer’s instructions. The purified fragment was cloned into the pSW2 vector at the *Mlu*I and *Xho*I sites. Cells of *E. coli* DH5α were transformed with this recombinant plasmid and selected on LB medium containing 25 µg/mL chloramphenicol. The positive clones were confirmed by enzyme digestion and DNA sequencing.

### Real Time qPCR using SYBR Green I Dye

The primer pairs of selected genes for real-time qPCR were designed using Primer Express software (ABI, Applied Biosystems, San Francisco, America). PCR cycling was conducted using 7500 System SDS software in reaction mixtures with total volumes of 20 µL containing 1× SYBR Green I Universal PCR Master Mix (ABI), 0.5 µM each primer, 1 µL template. The thermal cycling protocol was as follows: initial denaturation for 10 min at 95°C, followed by 40 PCR cycles of denaturation at 95 °C for 15 s, and annealing/extension at 60°C for 1 min. Melt curve (dissociation stage) was performed by the end of each cycle to ascertain the specificity of the primers and the purity of the final PCR product. Real time qPCR assays were performed in triplicate for each sample, and a mean value and standard deviation were calculated.

### DNA Copy Number Determination

The copy number of SW1 RF DNA was quantified by published relative quantification methods [40], [41] with some modification. A pair of primer SW1RFRTFor/SW1RFRTRev were designed to quantify the copy number of RF DNA, as the primer pair was located in the attP site of SW1 which uses the RF DNA as amplification template specifically. As the ssDNA can also serve as the template for the primer amplification, the two-step quantification was performed to determine the copy number of RF DNA and ssDNA. In brief, the total DNA was used as a template for the first-round qPCR, the copy number equaled RF DNA plus ssDNA. The template for the second-round qPCR is total DNA treated with S1 nuclease, after the ssDNA was removed from the total DNA, the quantification result was the copy number of RF DNA. Finally, the copy number of ssDNA was calculated from the two round qPCR.

The RCN was determined using equation 1, considering different amplification deficiencies (E) and Ct values for the two target genes.


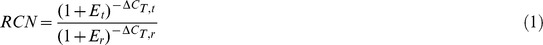


Where, E_t_ = amplification efficiency of target gene, E_r_ = amplification efficiency of reference gene, ΔC_T, t_ = targetC_T_ in sample−target C_T_ in calibrator, ΔC_T, r_ = reference C_T_ in sample−reference C_T_ in calibrator, target = SW1 RF DNA in WP3 total DNA, reference = *pepN* in WP3 total DNA, and sample = WP3 total DNA at 20°C or 4°C, calibrator = pSW3.

PCR amplification efficiency (E) was calculated according to the equation 2 as follows:


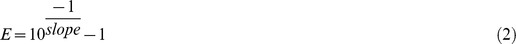


The relative standard curve was constructed placing the log value of the amount of DNA template on the x axis and threshold cycles on the y axis. In order to be consistent with the experimental conditions, we constructed the relative standard curve using WP3 total DNA as template. The curve was determined from five dilutions on conditions that r^2^≧0.99. The mass concentration of the template DNA was measured using a NanoDrop-2000 spectrophotometer (Thermo Fisher Scientific) and converted to the copy concentration using the equation 3 below: 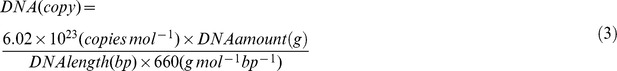



### mRNA Quantification with Real-time qPCR

The relative mRNA level of SW1 genes was quantified by the ΔΔC_T_ method [42]. In this method, the amount of target gene was normalized to that of the reference gene *pepN* whose expression remains stable under various conditions, relative to the calibrator. The transcription levels of the genes at 0 h were set as 1.

## Supporting Information

Figure S1 **Amplification efficiency assay of Q-PCR primers (standard curve).** Standard curves were constructed with serial 10-fold dilutions of the total DNA of WP3, ranging from 1×10^3^ to 1×10^7^ copies/µL. Each standard dilution was amplified by real-time QPCR using SW1RFFor/Rev and pepNRTFor/Rev in triplicate, and the determined CT values were plotted against the logarithm of their known initial copy number (n = 3). The data shown above represents average of triplicate assays.
(TIF)

Figure S2 **Relative mRNA levels of SW1 genes during the different growth periods of WP3 at 20°C and 4°C.** Relative mRNA levels of the two genes *fpsA* (A) and *fpsR* (B) at zero point were set as 1. Data shown above represents two independent experiments and the error bars indicate standard deviations from triplicate assays.
(TIF)
