# Supplementary figures and images for: Dynamic Modulation of DNA Replication and Gene Transcription in Deep-Sea Filamentous Phage SW1 in Response to Changes of Host Growth and Temperature
Source: PLoS One. 2012 Aug 1;7(8):e41578. doi: 10.1371/journal.pone.0041578 (PMC3411601; doi:10.1371/journal.pone.0041578)

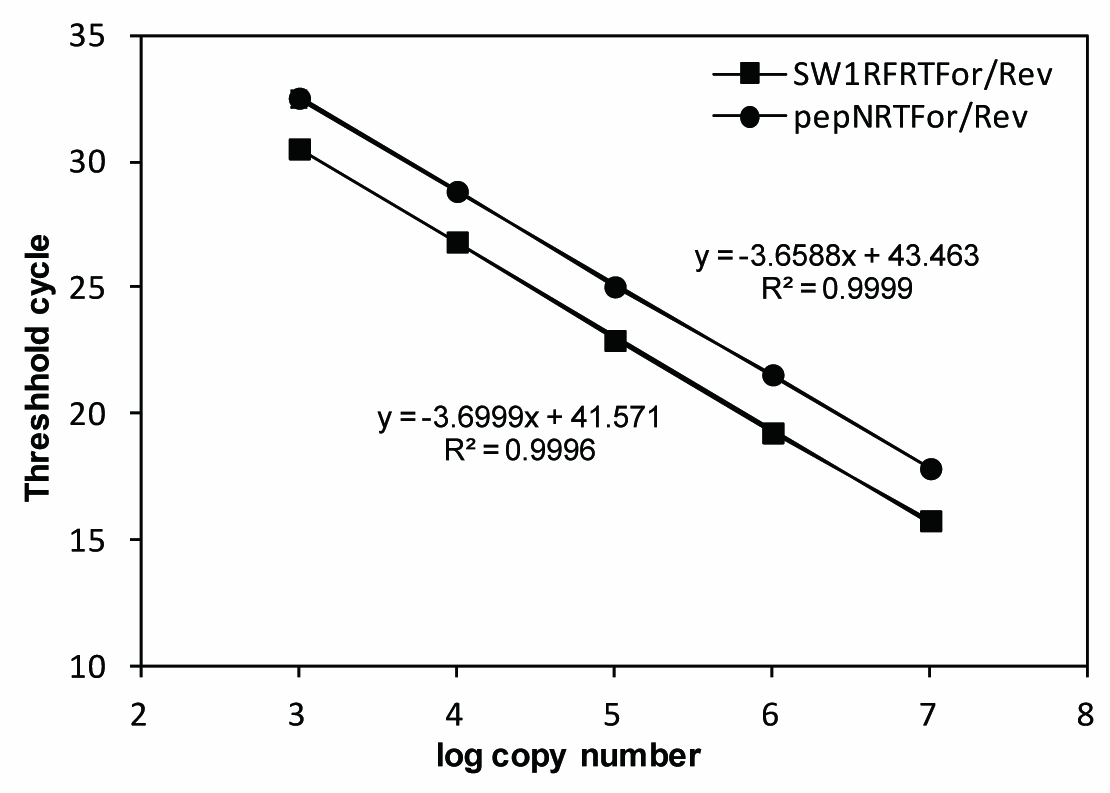

Supplement: Figure S1 — Amplification efficiency assay of Q-PCR primers (standard curve). Standard curves were constructed with serial 10-fold dilutions of the total DNA of WP3, ranging from 1×10^3^ to 1×10^7^ copies/µL. Each standard dilution was amplified by real-time QPCR using SW1RFFor/Rev and pepNRTFor/Rev in triplicate, and the determined CT values were plotted against the logarithm of their known initial copy number (n = 3). The data shown above represents average of triplicate assays. (TIF) [file pone.0041578.s001.tif]

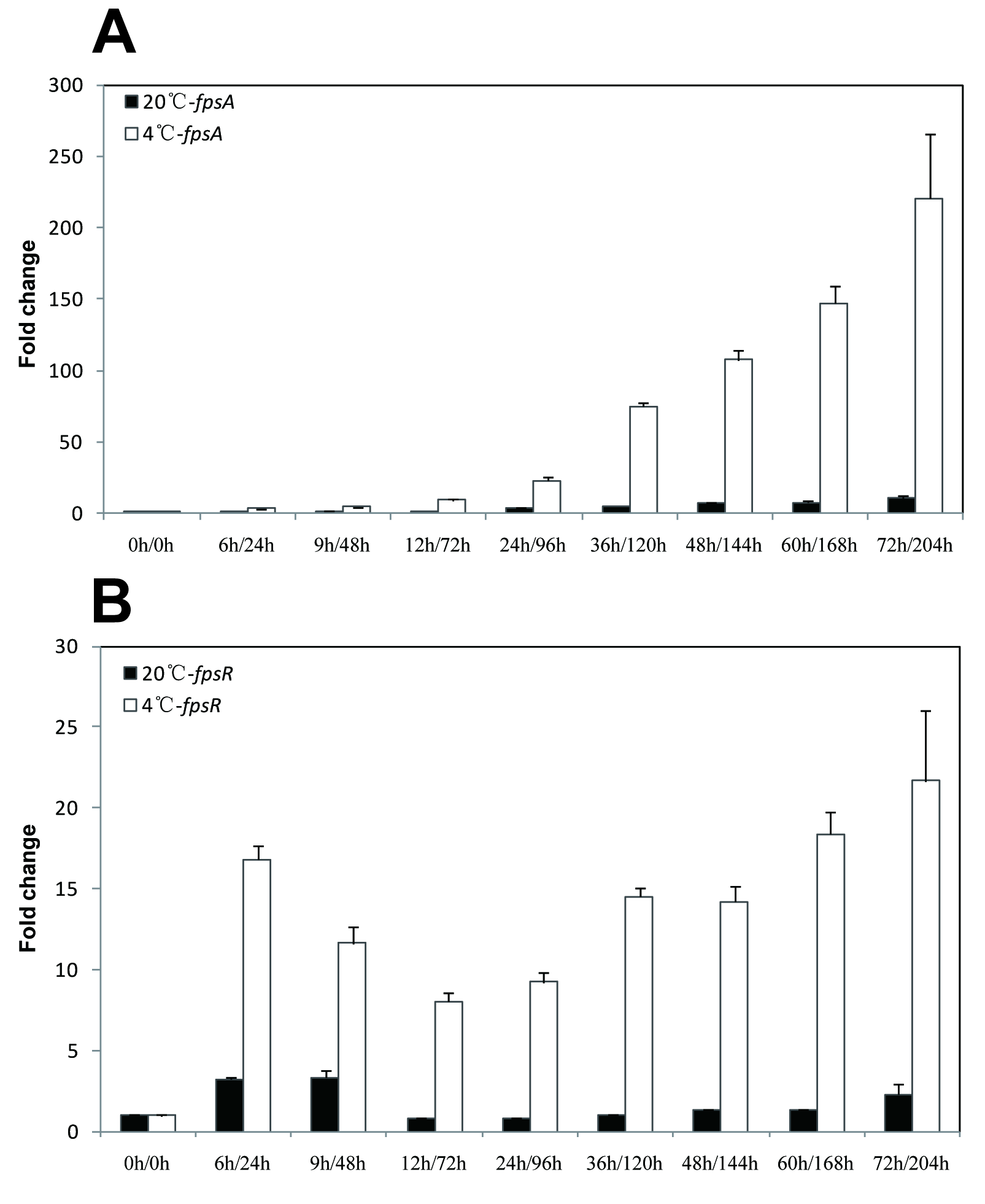

Supplement: Figure S2 — Relative mRNA levels of SW1 genes during the different growth periods of WP3 at 20°C and 4°C. Relative mRNA levels of the two genes fpsA (A) and fpsR (B) at zero point were set as 1. Data shown above represents two independent experiments and the error bars indicate standard deviations from triplicate assays. (TIF) [file pone.0041578.s002.tif]
